# Different Zn loading in Urea–Formaldehyde influences the N controlled release by structure modification

**DOI:** 10.1038/s41598-021-87112-2

**Published:** 2021-04-07

**Authors:** Amanda S. Giroto, Stella F. do Valle, Gelton G. F. Guimarães, Nicolai D. Jablonowski, Caue Ribeiro, Luiz Henrique C. Mattoso

**Affiliations:** 1 National Nanotechnology Laboratory for Agribusiness (LNNA), Embrapa Instrumentação, XV Novembro Street, CP: 741, São Carlos, SP 13560-206 Brazil; 2 Department of Chemistry, Federal University of São Carlos, Washington Luiz Highway, km 235, São Carlos, SP 13565-905 Brazil; 3 Agricultural Research and Rural Extension Company of Santa Catarina, 6800 Highway, Antônio Heil, Itajaí, Santa Catarina 88318112 Brazil; 4 Forschungszentrum Jülich GmbH, Institute of Bio- and Geosciences, IBG-2: Plant Science, 52425 Jülich, Germany

**Keywords:** Structural materials, Composites

## Abstract

Nitrogen fertilization has been a critical factor for high crop productivity, where urea is currently the most used N source due to its high concentration and affordability. Nevertheless, urea fast solubilization leads to frequent losses and lower agronomic efficiency. The modification of urea structure by condensation with formaldehyde has been proposed to improve nutrient uptake by plants and to reduce environmental losses. Herein we show that the co-formulation with Zn strongly modifies the N release (in lab conditions) and, more important, the Zn source—ZnSO_4_ or ZnO—has a critical role. Urea–formaldehyde (UF) served as a matrix for the zinc sources, and chemical characterizations revealed that Zn particles influenced the length of the polymeric chain formation. Release tests in an aqueous medium showed that the UF matrix favors ZnO release and, on the other hand, delays ZnSO_4_ delivery. Soil incubation with the fertilizer composites proved the slow-release of N from UF, is ideal for optimizing nutritional efficiency. Our results indicated that the ZnO-UF system has beneficial effects for both nutrients, i.e., reduces N volatilization and increases Zn release.

## Introduction

Boosts in modern agriculture are crucial to manage the increasing demand for food and other agro-products with the current trend of world population rapid growth. Chemical fertilizers have been essential to increase global agricultural production by approximately 50% over the last decades^1,2^. Most technological advances are related to N-based fertilizers, especially urea, the most commonly used N-fertilizer worldwide. Due to its high N content and safe handling, it is the least expensive fertilizer in terms of transportation cost per unit of nutrient and affordable to most farmers^3,4^. Unfortunately, its high water solubility leads to N losses, especially by volatilization, exceeding 50% of the total N applied and resulting in low fertilizer use efficiency^1,2,5,6^.

In this context, slow-release fertilizers (SRFs) have been shown as a promising approach to manage losses and improve nutrient use efficiency^2^. Urea–formaldehyde (UF) is one of the most common and the first group of products developed for SRFs fertilizer^7–11^. UF fertilizers are based on condensation products, mainly comprised of urea–formaldehyde polymers with different polymerization degrees^12^. UF is hydrolyzed by microorganisms in soil into ammonium, carbon dioxide, and water, allowing N absorption by plants. It also leads to complete compound degradation, i.e., an environmentally-friendly approach^13^. Besides all the positive points of UF fertilizers, their global market and production have been falling. Researchers argue that UF macromolecules' structure and crystallinity interfere in the material's short term biodegradation, being the main reason for the fertilizer industry to avoid this kind of product. The UF preparation can be modified using different processes to manage this aspect, e.g., solid-state processing, which abstains the use of extra reagents^8^; use of lower molar ratio between formaldehyde and urea producing shorter polymer chains and also urea units at the end of the chain; and reduction of hydrogen bond formation between UF chains to reduce crystallinity, thus improving the slow-release property^14^. The addition of some particles in the UF fertilizers during processing can reduce crystallinity by disturbing the regular UF molecular arrangement. In this sense, the introduction of a micronutrient source into the UF materials is beneficial. Some studies have indicated that the combined application of Zinc (Zn) and N fertilizers, for instance, promotes better nutrient absorption in crops for both elements. We propose that the micronutrient should be incorporated in the macronutrient fertilizer structure, as many fertilizer industries have been trying to do^15–17^.

Nevertheless, as much as crops require Zn in small quantities, the element is necessary for plant growth and survival as an essential constituent of many enzymes and proteins. Zinc deficiency is especially concerning in places with high cereal consumption in diets, reflecting directly in human nutritional health problems^4,18^. Zn is available for fertilization as zinc sulfate (ZnSO_4_), zinc carbonate (ZnCO_3_), zinc nitrate [Zn(NO_3_)_2_], zinc chloride (ZnCl_2_), and zinc oxide (ZnO)^19^. ZnO has the highest Zn content, i.e., 80% Zn/ZnO (w/w), making it the most cost-effective, although zinc sulfate is more often used due to its high solubility^20,21^.

Thus, herein we demonstrate that the Zn loading in UF structure affects N and Zn release in different forms depending on the Zn source. We have prepared slow-release fertilizers from UF with the addition of two sources of Zn (ZnO and ZnSO_4_), using the melting mix process strategy. This process is environmentally-friendly, easy to operate, and suitable for industrial production. The effects of the Zn sources in particle loading were thoroughly investigated using FTIR-ATR, TG/DTG, and ^1^H-, ^13^C-NMR, to verify changes in the morphology and structure that could influence their nutrient release profile. The dynamics of both N and Zn were investigated by release in water medium tests, and a release test of N in soil. In addition, a poor soil (with low N holding capacity and high Zn leaching capacity) was chosen to monitor the volatilization losses in lab conditions.

## Results and discussions

**Figure 1:**
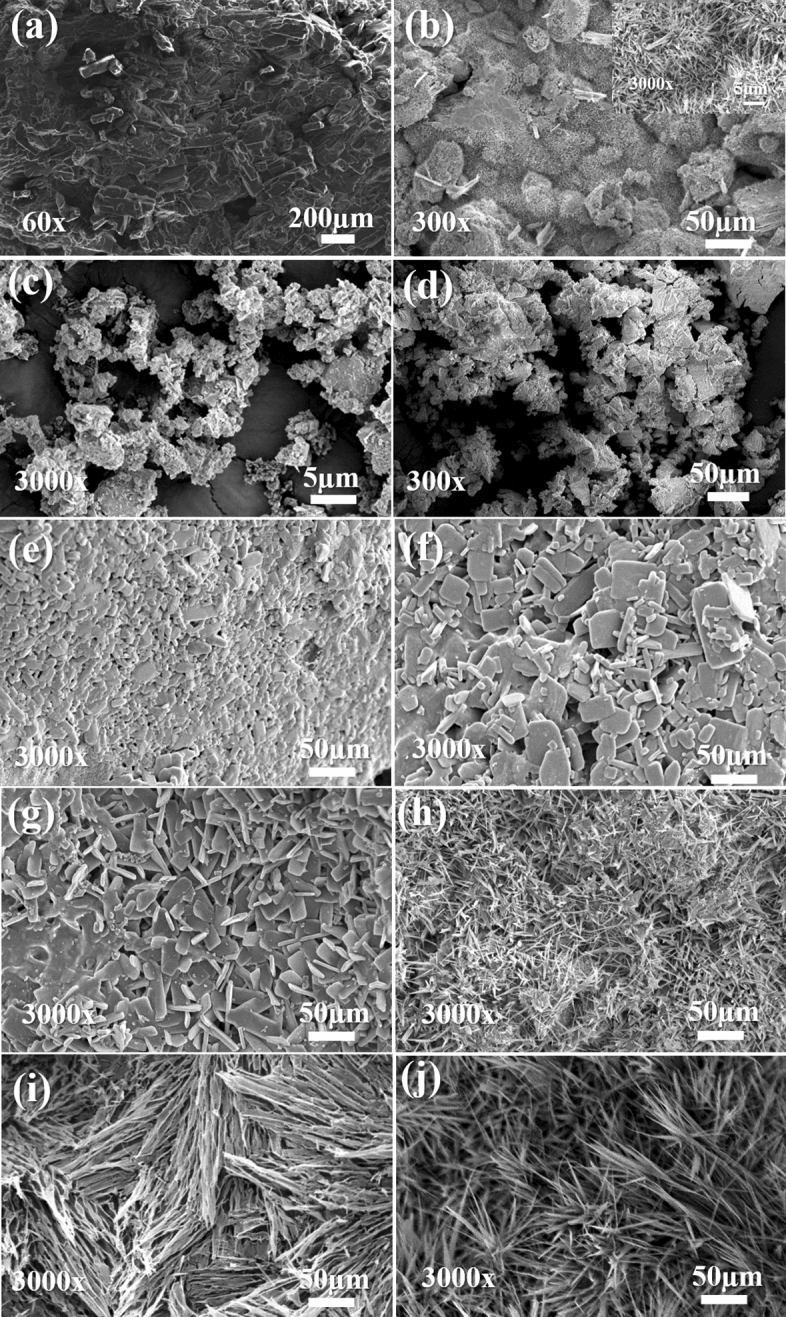
SEM images with its magnifications of (**a**) pure urea, (**b**) pure polymer UF, (**c**) ZnO, (**d**) ZnSO_4_, (**e**) UFZO 0.5, (**f**) UFZO 1, (**g**) UFZO 2, (**h**) UFZS 0.5, (**i**) UFZS 1 and (**j**) UFZS 2. (UF = pure polymer, UFZO = composites with ZnO and UFZS composites with ZnSO_4_).

Scanning electron microscopy (SEM) shows that urea is composed by agglomerated particles higher than 200 µm (Fig. 1a). The reaction with paraformaldehyde (UF) resulted in a heterogeneous morphology (Fig. 1b), with uneven surfaces covered by needle-shaped crystals, corresponding to the urea–formaldehyde fraction^22,23^. A new morphology is seen for the composites UFZO 0.5, 1, and 2 (Fig. 1e–g). ZnO probably acted as a catalyst in urea polycondensation, changing the polymeric structure to rectangular crystals (5–50 µm). These should not be ascribed as pure zinc oxide (Fig. 1c) or pure UF polymer due to the morphology differences. It indicates that the polymer is being nucleated by ZnO particles' surface, as seen by the good dispersion in EDX (Fig. S1, Supplementary Information). In contrast, the composites UFZS 0.5, 1, and 2, (Fig. 1h–j) present needle-shaped crystal formation, closer to pure UF, but with a superior size (superior to 50 µm), which is associated also to the dispersion of the ZnSO_4_ particles over the polymer (Fig. S1, Supplementary Information). Possibly, ZnSO_4_ is partly solubilized during processing, by the water that is released during the reaction of urea and formaldehyde. After solubilization, zinc sulfate presents a higher dispersion throughout the polymer, and precipitates as needles during the drying process. Therefore, ZnSO4 has acted as an electrostatic dispersing agent instead of a particle where the polymer is being nucleated.

**Figure 2:**
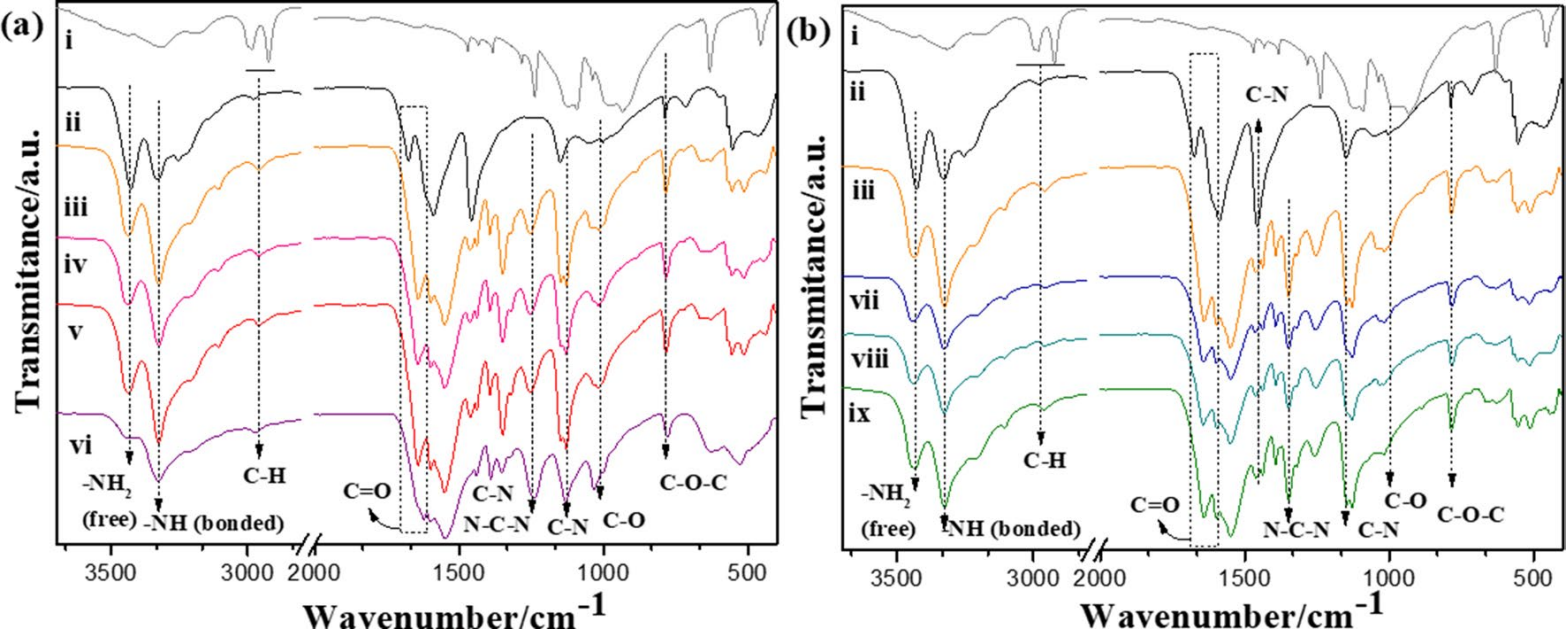
Normalized FTIR spectra in (**a**): (i) paraformaldehyde, (ii) urea (iii) pure polymer UF, (iv) UFZO 0.5, (v) UFZO 1, (vi) UFZO 2 and in (**b**): (vii) UFZS 0.5, (viii) UFZS 1 and (ix) UFZS 2.

The typical FTIR spectra of ZnO, ZnSO_4_, UF, UFZO, and UFZS are shown in Fig. 2, Fig. S2 (inset) and Table S1 (peak assignment). The identification of free NH_2_ groups shows that all composites have a polymer structure like urea:urea–formaldehyde as observed by Giroto et al.^8^. Thus, the materials have a mixed structure attributed to mono- or di-(hydroxymethyl) ureas, unreacted urea and linear polymerized urea chains. The region from 3350 to 3450 cm^−1^ is related to hydrogen-bonded O–H and N–H, mainly attributed to monomers such as water and formaldehyde, whose O–H group may form H bonds with reactive functional groups such as CH_2_OH, NH_2_, and N–H^24^. A major broad peak is seen for UFZO 2 (Fig. 2a) compared to the others. No residual paraformaldehyde was detected in the polymers^25^. Other characteristic peaks are amide I, II, and C=O, which were shifted to lower frequencies for both pure UF and all composites. The dislocation strongly indicates a partial hydrogen-bond (H-bond) formation between C=O and N–H groups when the urea–formaldehyde polymer is formed. It is possible that the samples have partial hydrogen bonds due to the C=O bond with or without H-bonds. A high concentration of methylene-bridge bonds (N–CH_2_–N) was detected^26^. These bands, together with the strong bands of C–N, C–O stretching in hydroxymethyl urea, and methylene ether linkage C−O−C, prove the formation of the structure of multiple methyl urea in the polymeric chain^14,27,28^. UFZO 2 was the only material to differ from the others, especially at 1700–1445 cm^−1^, very likely due to a higher H-bond formation between the multiple hydroxyethyl urea chains with different size lengths.

**Figure 3:**
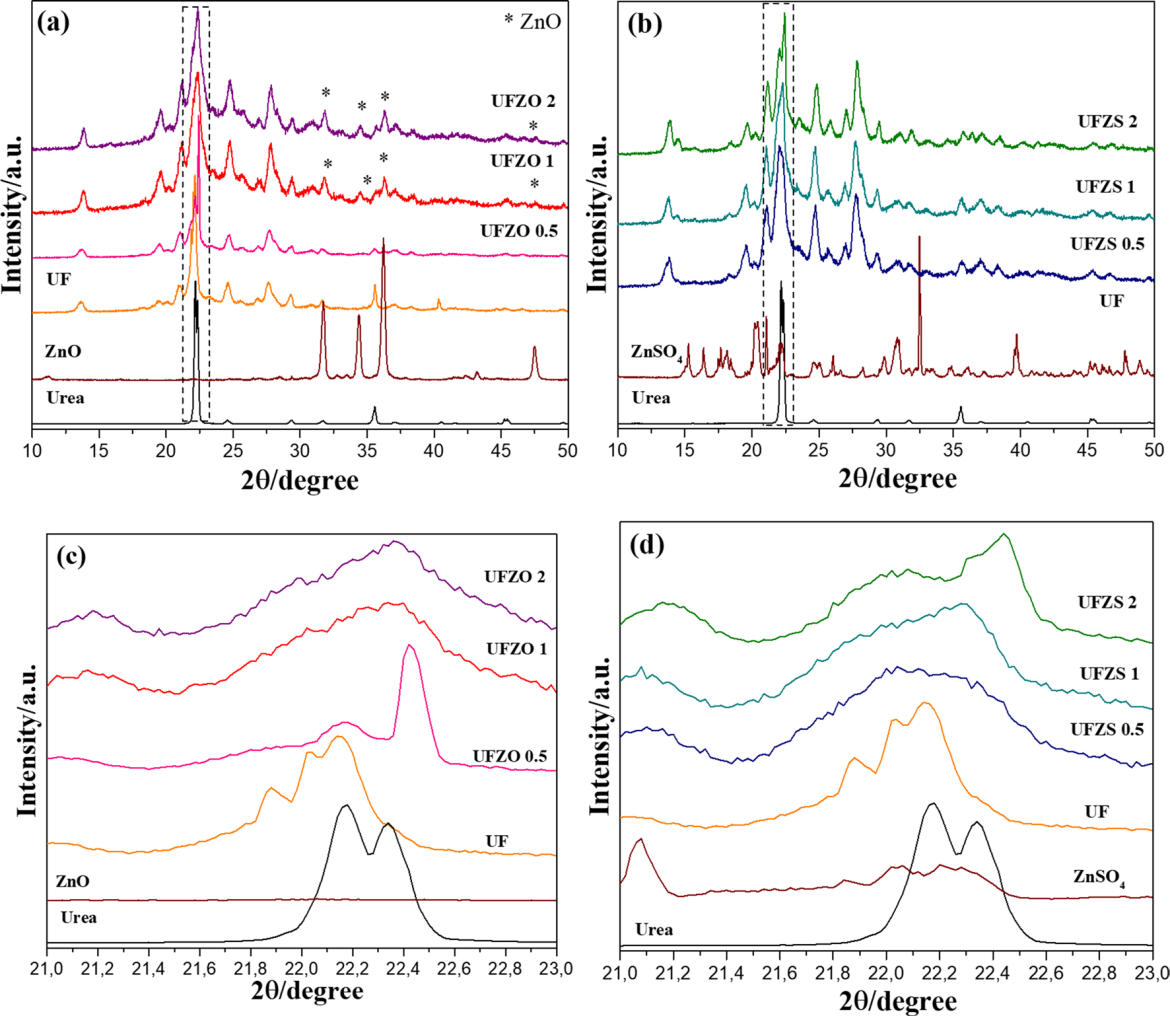
Normalized XRD pattern of Ur and UF with (**a**) ZnO, UFZO 0.5, UFZO 1, UFZO 2, (**b**) ZnSO_4_, UFZS 0.5, UFZS 1 and UFZS 2 (**c**, **d**) amplification the area at 2θ = 21–23°.

XRD patterns were conducted to study the crystalline structures of the samples. As shown in Fig. 3a, the existence of crystalline regions in UF is confirmed, possibly belonging to hydroxymethyl urea crystallized by H bonding during aging^29–31^. Peaks of ZnO were identified in the composites and no modification in UF pattern was seen, which indicates that the load did not change the UF crystalline phase. The same crystallization behavior was observed in Fig. 3b for UFZS composites. However, no signal of crystalline ZnSO_4_ particles was detected. In this case, the water released during urea polycondensation with formaldehyde might have solubilized embedded ZnSO_4_. In both composites, an increase on polymer signals is verified according to the Zn ratio. A broad peak in the range 2θ=10–30°, attributed to the amorphous region in UFZO 1, UFZO 2, UFZS 0.5, UFZS 1 and UFZS 2, suggests ramification or cross-linking during the condensation. Analysis of the amplified area at 21–23° (Fig. 3c,d) indicates the breakdown of the urea crystallinity of the composites UFZO 1, UFZO 2, UFZS 0.5, UFZS 1, and UFZS 2. It evidences the interactions of ZnO and ZnSO_4_ with UF. For UF, peaks at 22.2° and 22.4° of urea shifted to 21.9° and 22.2°, respectively, very likely due to the hydroxyethyl urea chains. UFZO 0.5 was the only one to maintain a crystalline peak of pure urea at 22.4°.

**Figure 4:**
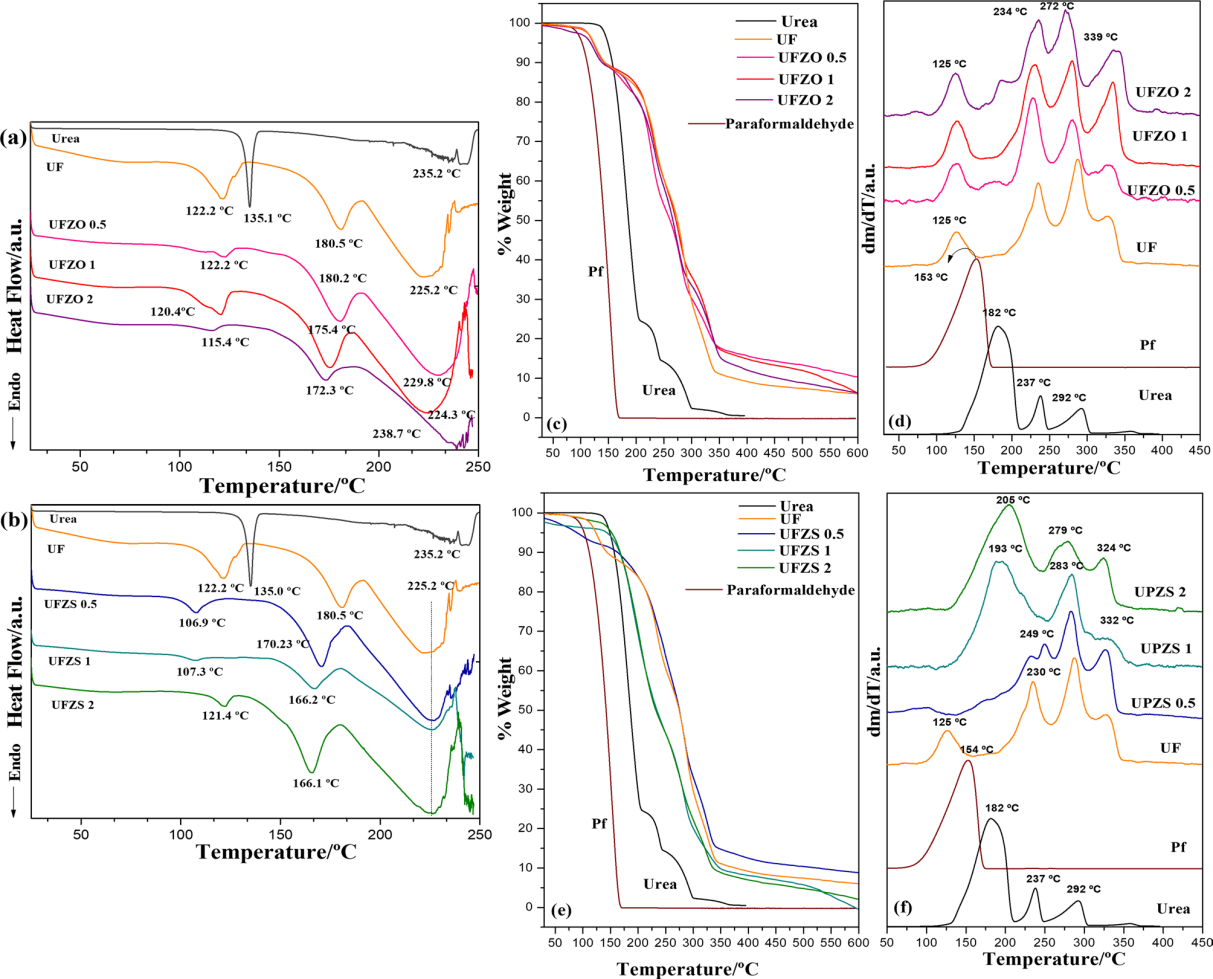
Termal analysis: DSC curves of urea, UF, (**a**) UFZO composites, (**b**) UFZS at a heating rate of 10 °C/min and (**c**–**f**) TGA of urea, UF, (**c**) UFZO composites, (**d**) UFZS obtained by TGA and expressed as the derivative (DTG) (**e**, **f**).

The urea-paraformaldehyde reaction was investigated by thermoanalytical methods. Figure 4 shows the DSC thermograms for UF materials. The endothermic event of urea melting (135 °C) shifted to lower temperatures (from 115 to 122 °C) in reacted materials, indicating the melting of oligomeric fractions or decomposition of bridges^32^. This peak should not be ascribed as paraformaldehyde depolymerization/volatilization (144 °C), since no residue was detected in FT-IR (Fig. 2)^7^. An endothermic event at 166–180 $^\circ$C can be seen, probably related to the melting of oligomeric fractions with methylene-bridges. The endothermic peak related to methyl urea bonds in UF (180.6 $^\circ$C) has shifted to lower temperatures. DSC analysis showed that Zn sources might play a role in catalyzing the thermal degradation, with endothermic peaks shifted to lower temperatures, indicating lower thermal stability. The complete decomposition of the compound occurs over 200 °C.

Comparing DSC results with thermogravimetry (TG and DTG) (Fig. 4c–f), it is possible to identify that urea degrades in three stages (peaks at 182, 237, 292 °C), and paraformaldehyde has a single stage (154 °C). The UF profile shows that the endothermic peak in DSC from 115 to 122 °C corresponds to a degradation mechanism, thus confirming the correlation to bridge breaking in oligomeric fractions. The next three stages (centered at 235, 287, and 330 °C) are probably related to the loss of functional groups of different chemical natures and energy costs^23^. UFZO composites behave very closely to pure UF; however, in the 2nd degradation stage it shifts to lower temperatures, suggesting the breakdown of the H-bonds. UFZO 2 (Fig. 4c) has an additional degradation event (183 °C) suggesting chains with intermediary lengths. The residual mass increases according to the Zn content. It supports that Zn has acted as a catalyst for breaking chain interactions. Nevertheless, UFZS materials have gradual decomposition depending on the polycondenzation degree. These composites show only 3 stages of degradation instead of 4, starting over 190 °C. UFZS 0.5 is closer to pure UF, while UFZS 1 and 2 had their 1st stage dislocated to lower temperatures with a major area of degradation compared to the subsequent events. As seen in XRD, ZnSO_4_ may be intercalated to the UF, changing the crystallinity and reducing the thermal stability. The residual UFZS was also dependent on the ZnSO_4_ content. Each Zn source led to different thermal behaviors, probably due to their different effects in crystallization.

**Figure 5:**
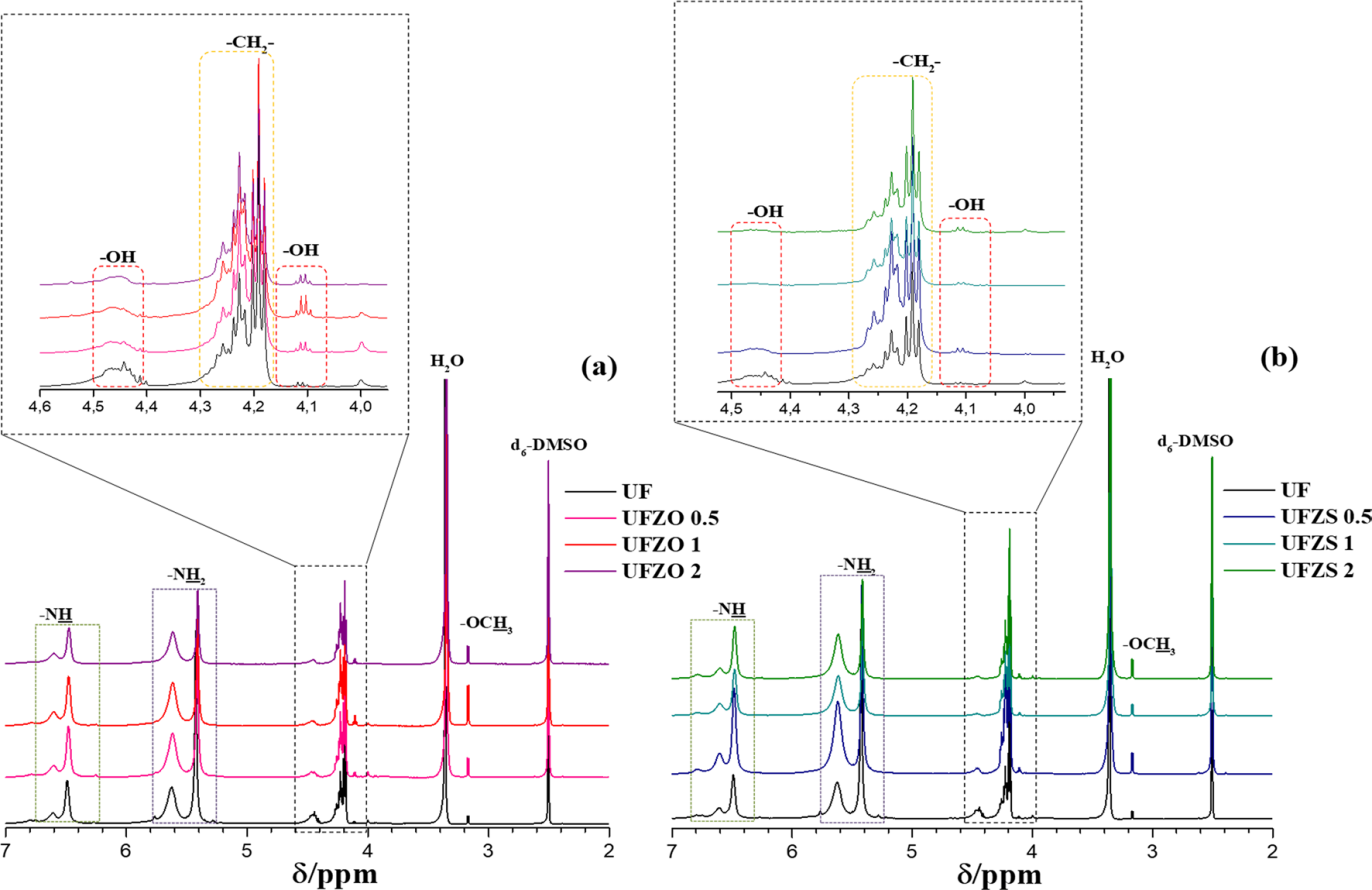
^1^H NMR spectra of UF and composites (**a**) UFZO and (**b**) UFZS solubilized in d_6_-DMSO.

**Figure 6:**
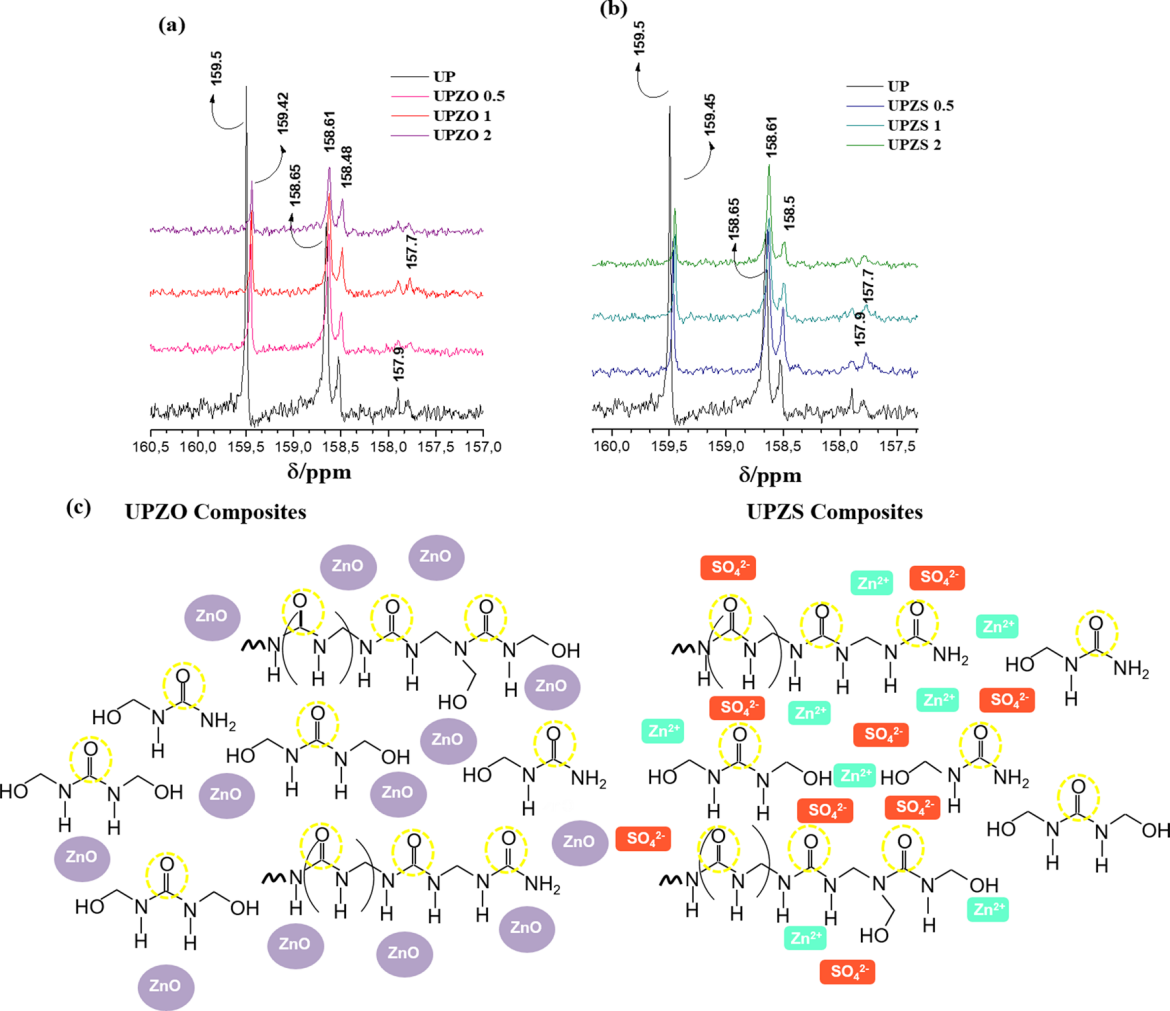
Amplification spectra of ^13^C NMR 160–157 ppm of composites (**a**) UFZO and (**b**) UFZS solubilized in DMSO—d_6_ and molecular structure proposed for the interaction of Zn particles with the polymers as well as the different medium of carbonyl groups. Molecular structure created using software Microsoft PowerPoint 2016 version.

The reaction extension and the effect of Zn sources has been verified by NMR techniques. ^1^H- and ^13^C- NMR and are shown in Figs. 5 and 6. Figure 5 a and b show the full spectra of ^1^H NMR from 7.00 to 2.00 ppm. There are substantial regions of monosubstituted amide (–CONHCH_2_–) at 7.00 − 5.80 ppm, nonsubstituted amide (–CONH_2_) at 5.80 − 5.40 ppm, hydroxyl (–OH) at 5.30 − 5.00 ppm, and methylene (–CH_2_–) signal at 5.00 − 4.10 ppm^29^. All materials have two signals at 6.83 and 6.60 ppm assigned to mono- and di-(hydroxymethyl) urea (Fig. S3 b). These signals confirmed the expected H-bond formation with the (–CONHCH_2_–) groups, as observed in the FTIR results. Multiple peaks at 5.61 indicate various chemical environments attached to the terminal –CONH_2_ group. Only −CONH_2_ attached to the smaller oligomer chains (Fig. S3 c) tends to have a higher chemical shift due to the inductive effect. Various oxygen atoms along the oligomer backbone could attract electron density from the hydrogen of −CONH_2_, since UF has a small peak at 5.76 ppm. For other materials as UFZO and UFZS composites, the higher intensity of the signal at 5.61 ppm implies that all materials had a proportion of the –CONH_2_ group attached to the long chains than that to the short ones. The strong peak at 5.41 ppm is assigned to –NH_2_ group of free urea, indicating the type of urea:urea–formaldehyde polymer structure. Multiple signals (4.52 − 4.15 ppm) are assigned to (–CH_2_–) methylene in (–NHCH_2_NH–) and in hydroxymethyl (HOCH_2_NH −) as illustrated in Fig. S3 d. A shoulder at 4.46 ppm is due to the presence of oxygen in the groups, which became smooth for the composites compared to pure UF, indicating that Zn guided the urea condensation to better dispersion, as shown in Fig.S3 c. It suggests that the formation of long UF chains is suppressed in composites. The increase of -OCH_3_ peak (3.16 ppm) (obtained by reaction of formaldehyde at the end of the polymer chain) in composites suggest that there are more terminal groups, thus, smaller chains, which supports the proposed Zn effect. The hydroxyls remain in all materials as evidenced by the signals from 4.0 − 4.12 ppm, further corroborating with the suggested formation of hydroxyl methyl urea structures along the main chain of urea-formaldehyde^26^. Urea–formaldehyde polymers cannot be described by a single formula^33^ and all analyses were done to approximate as much as possible to the real expected compound.

^13^C NMR is shown in Fig. 6, where the signals for urea carbonyls are assigned according to the substitution differences. No unreacted urea is seen in all materials, confirming that all urea was consumed in the polycondensation. The chemical assignments between 160 and 156 ppm are attributed to mono-, di-, and trisubstituted amides. Furthermore, the C=O signals at 159.5 ppm indicate the formation of H—bonds as shown in Fig. 6c, and it agrees to the ^1^H NMR and FTIR^27,29^. Di-substituted urea appears at 157–158 ppm and monosubstituted urea at 158–159 ppm^28^. All carbonyl signals shifted in due to Zn particles that disturb the carbon nucleus and modify its resonance. Therefore, all results indicate the formation of linear oligomers (with different lengths), and the formation of H-bonds between C=O and N–H groups in this kind of structure, leading to crystallization as verified by XRD pattern.

**Figure 7:**
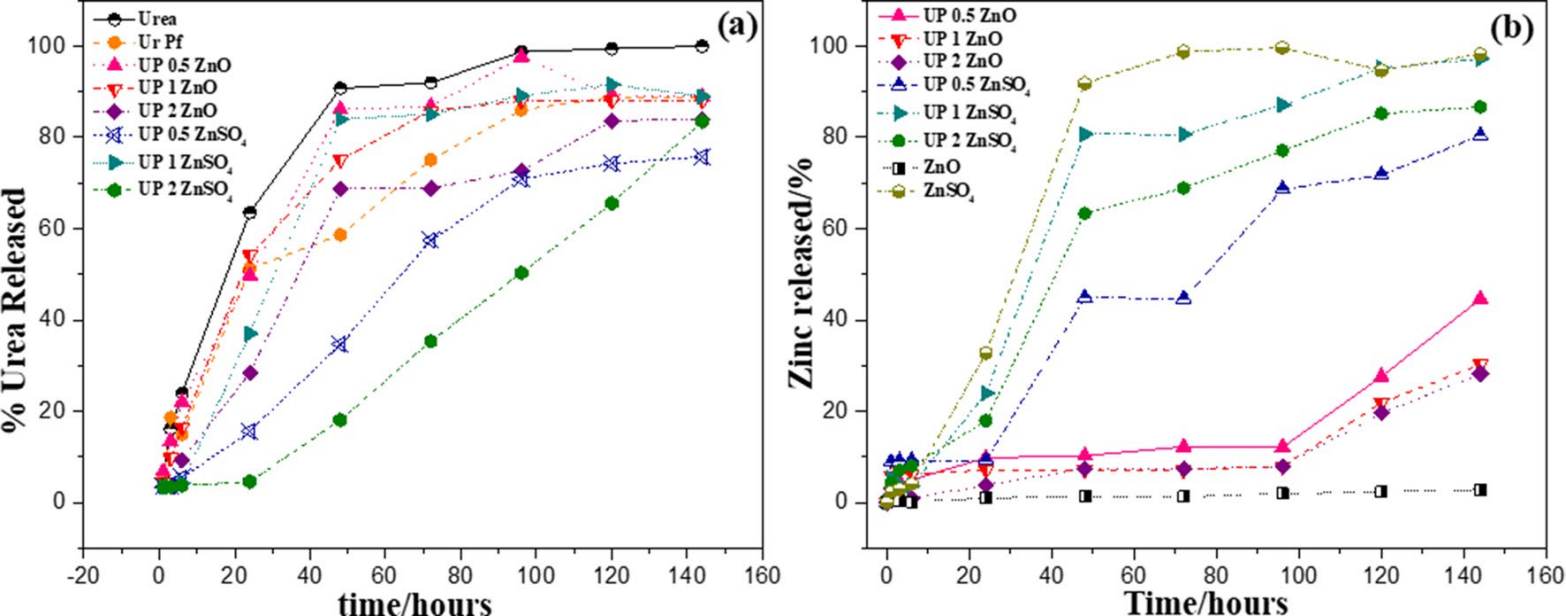
Release rates for (**a**) urea species and (**b**) zinc of materials cultivated in still water.

Figure 7a illustrates the urea release in water of pure urea and UF compared to all composites at 25 °C. It can be seen that almost all materials release more than 25% of the N content within 24 h. Some commercial urea brands show some controlled release behavior alone, as observed in Fig. 7. A small portion of formaldehyde might have been added to help the granulation process. Interestingly, when analyzing the FTIR of pure urea, the presence of this compound can be seen by the enlargement of the NH stretching of urea as well as the duplication of NH signals, which also indicate some interaction of NH of urea and OH band of formaldehyde form in the granule. Therefore, UF polymers presented a small difference compared to pure urea in the release test in water medium. As discussed, it is expected that the materials have a more controlled release compared to pure urea, due to the formation of small molecules of substituted urea, as observed in our experiments. About 20–30% of the urea has remained in the materials after 140 h, while pure urea is completely solubilized. UF fertilizers are already recommended to fertilize perennial plants, such as forests, fruit plants, and lawns, which require long N release^30^. A new range of crops could be targeted to N fertilization with the studied materials. The different profiles of release from the composites and UF is due to the intercalated Zn particles. Figure 7b shows Zn release behavior from the composites and pure sources (ZnO and ZnSO_4_). In contrast to ZnO, which has no solubilization in the period of the experiment, UFZO materials released 30–40% of Zn after 140 h. The dispersion effect increases the ZnO solubilization, in a similar effect as observed for hydroxyapatite powders in urea^34^. On the other hand, ZnSO_4_ results in a controlled-release in the composites, instead of the fast delivery observed from the highly soluble pure ZnSO_4_ salt. The increased interaction of ZnSO_4_ with the UF structure can explain this behavior, as seen by the thermoanalysis and FTIR. It also corroborates that the ZnSO_4_ salt is solubilized during synthesis and re-precipitates within the UF structure, being highly intercalated and dispersed in the matrix and acting as physical and chemical barrier for hydrogen bond formation.

**Figure 8:**
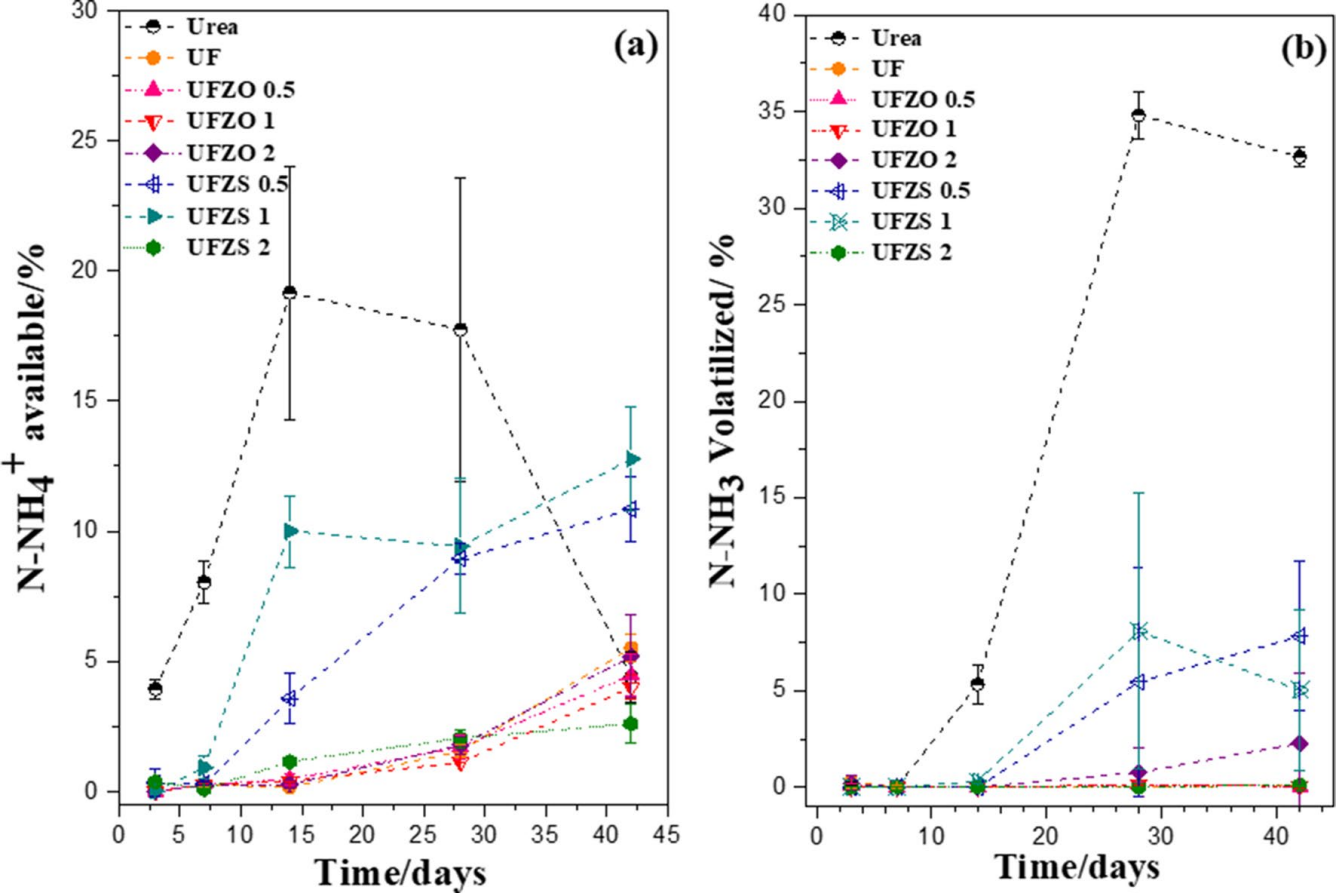
N recovery from the fertilizers as (**a**) N–NH_4_^+^ available and (**b**) N–NH_3_ volatilized for each fertilizer treatment over the 42-day incubation period.

The incubation in soil for 42 days reveals the behavior of N in real conditions and the effects of each Zn source, as shown in Fig. 8. In this experiment, N transformation was monitored from urea hydrolysis, assessing the available N-NH_4_^+^ in soil and the loss of N by NH_3_ volatilization. N-release rate from UF and the composites was slower in the first 7 days and then gradually raised up, while pure urea displayed a fast initial release and started to decline after 14 days. UFZS 0.5 and 1 showed the highest NH_4_^+^ values after 14 days among the composites, but still lower than pure urea. This suggests that ZnSO_4_ is favoring the N release from the UF structure. However, the same result was not observed for UFZS 2, which had no release of urea in water medium (Fig. 7a). Pure UF and UFZO composites also presented low N release in soil (<5% of total N), indicating a need for longer periods (more than 42 days) to fully release the structural N, typical for slow-release fertilizers. UF presents a lower solubility compared to pure urea as its structure is more complex, with different chain lengths and substituents, slowing down the hydrolysis rate^35^.

Therefore, most of the N in the composites could be released with longer incubation time. The first N released to soil is possibly from low length compounds (mono and hydroxymethyl ureas or mono- and di- substituted ureas) while the longest release is due to hydrolysis of longer chains. Zn reduces the interaction between chains, especially ZnSO_4_ by its lower pH compared to ZnO. This behavior is verified by Zhang et al.^11^, in their NPK composites. With the highest profile of NH_4_^+^ release from UFZS0.5 and UFZS1, they also achieved the highest volatilized NH_3_ values among the composites, with 7.5 and 5.5%, respectively, which was still lower than pure urea (35%) in 28 days.

Table 1 shows the N application in soil that has been lost (volatilized) as NH_3_ and is still available after 42 days of incubation. N-residual corresponds to the nutrient still in the fertilizer matrix or immobilized by the soil microbiota. Although a small fraction could be lost by the formation of other N oxides that were not measured, they were considered less important in the experimental context. The results confirm the low availability of N-NH_4_^+^ species after 42 days of urea incubation in soil. This low efficiency is attributed to the high NH_3_ volatility, which in this study was 32% by the end of the experiment^36–38^. As previously discussed, although urea provides high availability of N-NH_4_^+^ 14 days after incubation, the available N for plant absorption considerably reduces after 28 days (Fig. 8a). The reduction in N-NH_4_^+^ is a consequence of its transformation to NH_3_ and subsequent loss through volatilization. In this way, the availability of N from urea fertilizer is concentrated in a short period of time, which reduces the use/absorption by the plants that require the nutrient throughout their vegetative and reproductive cycles. This result highlights the low agronomic efficiency of the urea when applied to the soil in these conditions^35,36^. This is one of the reasons for the effort in the search for a fertilizer capable of supplying N in a more regular and constant way, that can be synchronized with the plants' demands.

**Table 1:** N recovery from the fertilizers as N-NH_4_^+^ available, and N-NH_3_ volatilized, after 42 days soil incubation. The data shows average values of each N fraction derived of total N applied to the soil as urea, UF or composites. (UF = pure polymers, UFZO = composites with ZnO and UFZS composites with ZnSO_4_). The estimative of N recovery percentage was calculated by the total N content present in the composites (10 mg of N) subtracted by the N content converted into ammonium or ammonia extracted from the soil (determined by colorimetric or titulation method).

Fertilizers	N-NH_4_^+^_available in soil_	N-NH_3 volatilizated_
	%	
Urea	4.40 b	32.7 d
UF	5.52 b	0.13 a
UFZO 0.5	4.47 b	0.05 a
UFZO 1	4.00 bc	0.05 a
UFZO 2	5.18 b	2.27 ab
UFZS 0.5	10.84 a	7.82 c
UFZS 1	12.77 a	7.41 bc
UFZS 2	2.60 c	0.13 a

*Values within a column followed by the same letter do not differ significantly (Tukey's test; P < 0.05) with n = 4 replicates.

Regarding this purpose, the N release profiles of the composites UFZS 0.5 and 1 indicate their potential use, considering the controlled N release, lower NH_3_ volatilization and greater NH_4_^+^ availability after 42 days of incubation, compared to pure urea (Fig. 8 and Table 1). By delivering N in a slower manner and avoiding its loss, the composites are less damaging to the environment and more efficient in the longer term. In addition, these materials provide the possibility of joint supply of N and Zn to plants, besides the effect of the interaction between urea–formaldehyde with Zn sources (ZnO and ZnSO_4_), which proved to be strategic to modulate the polymer chain growth and improve the Zn accessibility.

## Methods

### Materials

Urea (Synth, Brazil) and paraformaldehyde (Sigma-Aldrich, USA) (both chemical structures displayed in Fig. S3 a), ZnO and ZnSO_4_·7H_2_O (Synth, Brazil) were used for the materials' production. Urea and ZnSO_4_ salt were previously milled to a size range of ≤ 300 µm using a TE-330 hammer mill (Tecnal, Brazil). The ZnO powder was sieved in 300 µm to standardize the initial particle size.

### Preparation of materials

UF was prepared following the molar ratio of 1:0.5 between urea and paraformaldehyde. The reagents were processed using a torque rheometer (Polylab RHEODRIVE Rheomix mixer and OS4) under 60 rpm and 90 °C for 10 min to mix-melt the reagents. For the UF/Zn composites, all components were used as powders with standardized particle sizes of ~ 300 µm, previously mixed in plastic bags to achieve homogeneity before being submitted to the mixed-melting process, maintaining the same molar ratio of urea: paraformaldehyde as described. The Zn was added in varying proportions to the UF, namely 0.5, 0.7, and 1.45% (wt.%) of Zn for both ZnO and ZnSO_4_. After mixing, the samples were cured in an oven at 80 °C for 12 h and before storage all samples were classified using a sieve of < 500 µm. No float was observed during the synthesis once urea melting helped in aggregating all solids together, followed by the homogenization reached by the mixture in the rheometer machine. The name and the total percentage of nutrients are described in Table 2.

**Table 2:** List of named composites with their final composition.

Materials	Name	% Nutrient	
		N	Zn
Urea	Ur	45	–
UrPf 1:0.5	UF	40.71	–
UrPfZnO 0.5	UFZO 0.5	40.07	0.456
UrPfZnO 1	UFZO 1	41.40	0.732
UrPfZnO 2	UFZO 2	40.43	1.440
UrPfZnSO_4_ 0.5	UFZS 0.5	43.70	0.352
UrPfZnSO_4_ 1	UFZS 1	44.52	0.635
UrPfZnSO_4_ 2	UFZS 2	37.96	1.880

### Characterizations

The composites' morphologies were characterized by scanning electron microscopy (SEM) using a JSM6510 microscope (JEOL) using the secondary electron mode. Thermal analysis was conducted at the range of 25 °C to 600 °C using a Q500 analyzer (TA Instruments, New Castle, DE, USA) with a heating rate of 10 °C min^−1^ under nitrogen atmosphere. For the differential scanning calorimetry (DSC), samples were heated from 25 to 250 °C in a DSC Q100 (TA Instruments, USA) under nitrogen atmosphere. Fourier Transform Infrared was carried out in an FTIR spectrophotometer VERTEX 70 (Bruker Corporation) with ATR technique. Proton and carbon nuclear magnetic resonance (^1^H- and ^13^C- NMR) spectra were obtained on a 600 MHz Avance III HD Bruker spectrometer using dimethyl sulfoxide (DMSO-d_6_) as a solvent and tetramethylsilane (TMS) as the internal reference.

### Release tests in solution

The composites were submitted to test for both urea and Zn release as a function of time. The release rate in water was determined by adding the samples to beakers and gently stirring for 5 days using an orbital shaker at 50 rpm (Thermo scientific) and 25 °C. To determine the dissolution of urea and zinc, aliquots of 2 mL were collected and centrifuged (15 min at 14,000 rpm, MiniSpin Plus) at different time intervals (three times in the first day and after once a day), over 5 days. The maximum level of urea added in each experiment was the same (1250 mg L^-1^); that is, different mass values for the composites were calculated so that each experiment had the same amount of urea. For comparison, a test with pure urea, ZnO and ZnSO_4_ was also performed as a control experiment. The determination of urea concentration in solution was done by UV–vis spectrophotometry (Shimadzu-1601PC), according to the method of Tomaszewska and Jarosiewicz^39^, and Giroto et al.^8^. Zn release rate determination was performed using atomic absorption spectrophotometry using a part of the aliquots (PinAAcle 900 T- PerkinElmer). Thus, a curve of urea and zinc concentration in solution versus release time was obtained.

### Nitrogen mineralization in soil

Nitrogen transformations in soil to ammonium and ammonia volatilization were investigated in an Oxisol (Red-Yellow Oxisol which was collected at 20 cm depth at a pasture site in São Carlos, Brazil), which was characterized according to its physical and chemical aspects^40–42^. Details of the soil characteristics are provided in Table 3. The soil samples were air-dried and crushed to pass through a 2 mm screen before use. Soil samples (10 g) were incubated using an incubation system with the tested fertilizers at a 1000:1 g g^-1^ ratio of soil:N, placed in 125 mL polyethylene screw-cap bottles as Ur, UF and composites (UFZO 0.5, 1 and 2) or (UFZS 0.5, 1 and, 2), as described by Guimarães et al.^36^ and Giroto et al.^8^. Samples were incubated for 3, 7, 14, 28 and 42 days under controlled temperature (25 °C) and humidity (60% WHC).

**Table 3:** Chemical and physical properties of the studied soils.

Soil characteristics									
pH	CEC^a^	Organic C	Sand	Silt	Clay	WHC^b^	N	Zn	Urease activity
	(cmol_c_ kg^-1^)	(g kg^-1^)						(mg kg^-1^)	(mg N kg^-1^ h^-1^)
5.0	4.2	7.0	433	35	532	200	1.06	0.51	7.1

^a^CEC, cation exchange capacity.

^b^WHC, water-holding capacity.

Analyzes were performed after each 3, 7, 14, 28 and 42 days. Determinations were carried out via volatilization^43^ and the soil mineralization of N-NH_4_^+^^44^ followed the description used by Giroto et al.^37^. Volatilized NH_3_ was quantified by titration of boric acid with HCl (0.01 mol L^−1^). Mineral N produced during incubation was extracted by shaking the soil sample with 100 mL of KCl (l mol L^−1^) containing phenyl mercuric acetate (5 mg L^−1^) as a urease inhibitor (soil:solution ratio of 1:10). Afterwards, the suspension was stirred for 1 h and filtered through filter paper (diameter 12.5 cm Whatman no. 42 filter paper (GEHealthcare, Buckinghamshire, UK)). The resulting soil extract was stored in 100 mL polyethylene bottles at 5 °C. The ammonium (NH_4_^+^) levels in the soil extracts were determined by the colorimetric methods of Kempers and Zweers^44^. The contents recovered in each N fraction (NH_4_^+^ and NH_3_) were expressed as percentages in relation to the N added to soil in the form of urea or composite.

Analysis of variance (ANOVA) was done for the differences among treatments by total recovery as NH_3_ volatilized and exchangeable NH_4_^+^ after aerobic incubation and when the F test was significant, differences among treatments were compared by the Tukey test (P < 0.05).

## Conclusions

The results of this study showed that the formation of the UF polymers was affected by the loading of Zn in composites during the synthesis with no dependence on a specific Zn source. XRD analysis clearly showed the distinction in the crystallinity of the materials with the addition of Zn, which was also verified in the nutrients released in water medium, promoting the controlled release of nitrogen in all composites. Zn sources featured a different solubilization behavior in the release test. ZnSO_4_, a soluble source, had a controlled delivery due to its dispersion throughout the UF composite, as verified by XRD and SEM analysis. On the other hand, the low solubility of ZnO was enhanced in the composites, with a better performance after 4 days of water immersion, releasing 40% of Zn at 7 days. ^1^H- and ^13^C- NMR analyses showed the Zn particles have unsettled the arrangement of the polymer chains, which prevented the length growth of the polymer chains compared to the pure UF. This study proved the feasibility of the production and applications of UF loaded with Zn, as slow-release fertilizers or other products in agriculture, showing the beneficial effects for both nutrients, i.e., reduces N volatilization and increases Zn bio-availability.

## Supplementary Information

Supplementary Information
