# Supplementary material for: Different Zn loading in Urea–Formaldehyde influences the N controlled release by structure modification
Source: Sci Rep. 2021 Apr 7;11:7621. doi: 10.1038/s41598-021-87112-2 (PMC8027403; doi:10.1038/s41598-021-87112-2)
Supplement: Supplementary file 1 — Supplementary Information [file 41598_2021_87112_MOESM1_ESM.docx]

**Supplementary Information**

**Different Zn loading in Urea-Formaldehyde influences the N controlled release by Structure Modification**

Amanda S. Giroto ^1^, Stella F. do Valle,^1,2^, Gelton G. F. Guimaraes ^3^, Nicolai D. Jablonowski^4*^, Caue Ribeiro^1*^, Luiz Henrique C. Mattoso^1^

*^1^Embrapa Instrumentação, National Nanotechnology Laboratory for Agribusiness* *(LNNA), XV Novembro Street, CP: 741, Zip Code: 13560-206 São Carlos, SP, Brazil*

*^2^Federal University of São Carlos, Department of Chemistry, Washington Luiz Highway, km 235, Zip Code: 13565-905 São Carlos, SP, Brazil*

*^3^Agricultural Research and Rural Extension Company of Santa Catarina, 6800 Highway, Antônio Heil, Itajaí, Santa Catarina, 88318112, Brazil*

*^4^Forschungszentrum Jülich GmbH, Institute of Bio- and Geosciences, IBG-2: Plant Science, 52425 Jülich, Germany*

*Corresponding authors: n.d.jablonowski@fz-juelich.de; caue.ribeiro@embrapa.br

Table S1. FTIR bands related to bonds of the materials.

| **Bands** |  |  | **Wavenumber (cm^-1^)** | | | | | |
| --- | --- | --- | --- | --- | --- | --- | --- | --- |
|  | **Urea** | **UF** | **UFZO** | | | **UFZS** | | |
|  |  |  | 0.5 | 1 | 0.2 | 0.5 | 1 | 2 |
| ν-NH_2 (free)_ | 3425 | 3437 | 3437 | 3437 | 3437 | 3443 | 3443 | 3343 |
| ν-NH _(bonded)_ | 3327 | 3327 | 3327 | 3327 | 3327 | 3327 | 3327 | 3327 |
| νCO_free_ | 1676 | 1646 | 1646 | 1646 | 1646 | 1646 | 1646 | 1646 |
| δN-H +νC-N | 1588 | 1599 | 1599 | 1599 | 1599 | 1599 | 1599 | 1599 |

Figure S1. EDX images of composites (a) UFZO 0.5, UFZO 1, UFZO 2, and (b) UFZS 0.5, UFZS 1 and UFZS 2.

Figure S2. Normalized FTIR spectra in (a) and (c) amplification to 3500-3000 cm^-1^ and (b) and (d) 1800-1500cm^-1^ for (i) urea, (ii) pure polymer UF, (iii) UFZO 0.5, (iv) UFZO 1, (v) UFZO 2 and (vi) ZnSO_4_, (vii) UFZS 0.5, (viii) UFZS 1 and (ix) UFZS 2, (x) ZnO and (xi) ZnSO_4._


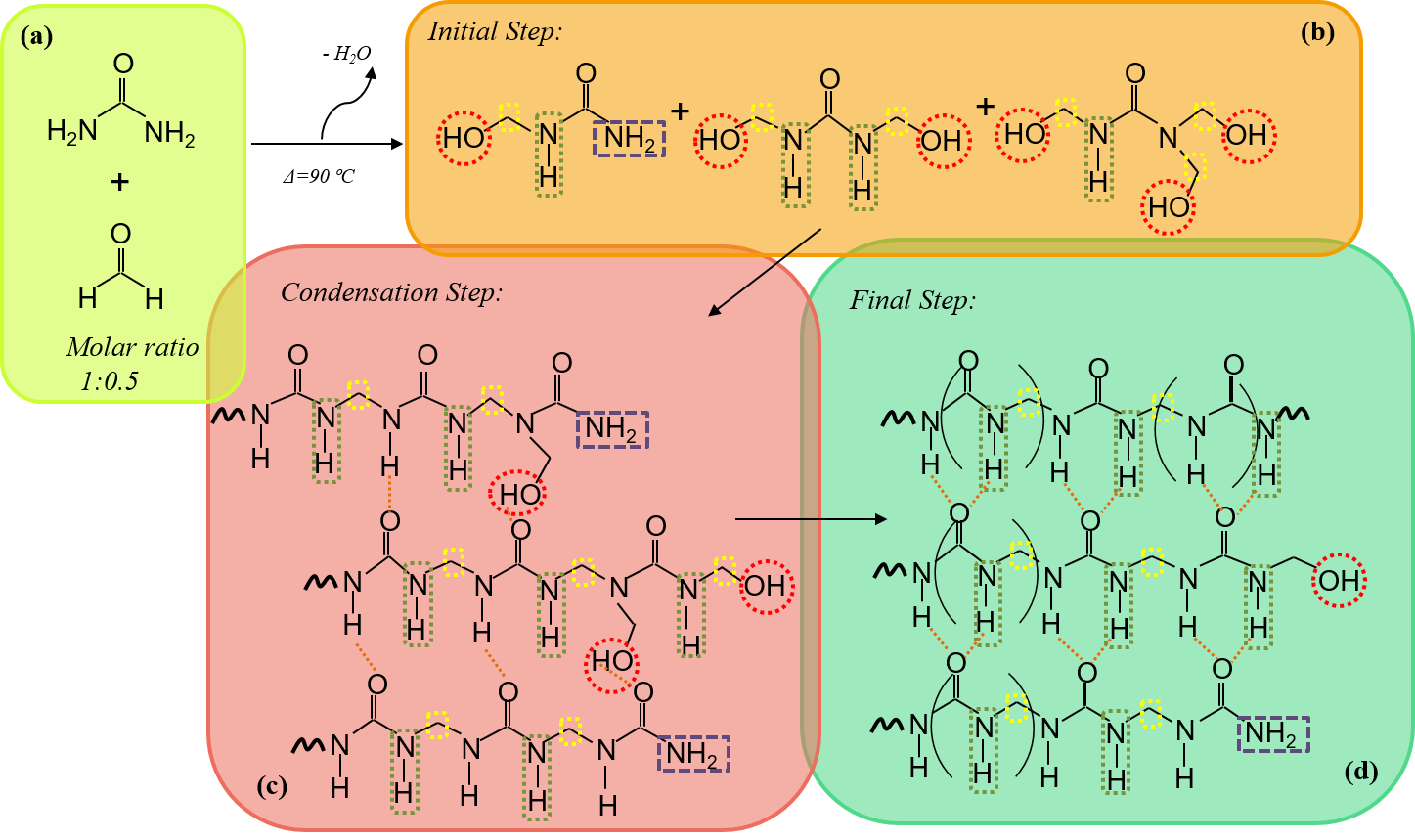


Figure S3. Schematic illustration of the possible chemical species corresponding to the ^1^H NMR Spectra: (a) urea and formaldehyde, (b) small molecules formed: mono, di- and tri hydroxylureas, (c) condensation step with hydroxyl groups and (d) cure step and H-bond formation. The illustration was made in the software Microsoft PowerPoint 2016 version.
